# Late presentation of HIV infection in the country of Georgia: 2012-2015

**DOI:** 10.1371/journal.pone.0186835

**Published:** 2017-10-30

**Authors:** Nikoloz Chkhartishvili, Otar Chokoshvili, Natalia Bolokadze, Maya Tsintsadze, Lali Sharvadze, Pati Gabunia, Natia Dvali, Akaki Abutidze, Tengiz Tsertsvadze

**Affiliations:** 1 Infectious Diseases, AIDS and Clinical Immunology Research Center, Tbilisi, Georgia; 2 Ivane Javakhishvili Tbilisi State University, Tbilisi, Georgia; University of Cyprus, CYPRUS

## Abstract

Late presentation for HIV care has important individual and population implications. The objective of this study was to explore the problem of late presentation in the country of Georgia. Data on adult persons newly diagnosed with HIV in Georgia between 2012 and 2015 were extracted from the national AIDS Health Information System. Late presenter was defined as a person diagnosed with HIV with a CD4 cell count <350 cells/mm^3^ or an AIDS defining illness regardless of the CD4 cell count in the six months after HIV diagnosis. Late presenter with advanced disease was defined as a person diagnosed with HIV with a CD4 cell count <200 cells/mm^3^ or an AIDS defining illness, regardless of CD4 cell count in the six months after HIV diagnosis. Among 2267 adults diagnosed with HIV in Georgia in 2012–2015, 1987 (87.6%) had CD4 cell count measured within 6 months of HIV diagnosis and were included in the analysis. Among them 1260 (63.4%) patients were classified as late presenters and 870 (43.8%) as late presenters with advanced disease. The proportion of late presenters declined from 71.1% in 2012 to 55.5% in 2015 (p<0.0001), while presentation late with advanced disease decreased from 56.6% in 2012 to 34.5% in 2015 (p<0.0001). Late presentation was most common among people who inject drugs (77.7%). Overall 186 patients died over the studied period. Mortality was higher both among late presenters (6.74 per 100 person-years vs. 1.08 per 100 person-years, p<0.0001) and late presenters with advanced disease (8.93 per 100 person-years vs. 1.34 per 100 person-years, p<0.0001). High prevalence of late presentation in Georgia reflects insufficiency in HIV testing services. Better testing strategies are needed to improve earlier diagnosis and disease outcomes.

## Introduction

HIV testing is considered as a gateway to HIV care, treatment and prevention. However, in many settings, coverage with HIV testing services is not adequate. UNAIDS estimates that only 57% of estimated 36.7 million people living with HIV (PLHIV) globally know their HIV status [1]. In addition, half of those diagnosed enter care late, defined as presenting to care with CD4 cell count <350 cells/mm^3^ or an AIDS defining illness within 6 months after HIV diagnosis [2–4]. Late diagnosis/presentation has serious negative consequences in terms of survival, resource use and onward transmission to others [5].

Georgia is an Eastern European nation with steadily growing HIV epidemic. Although the growth of the epidemic in Georgia has been slower than in the rest of the region, the number of new diagnoses has risen each year [6]. By the end of 2015 a cumulative 5412 cases of HIV infection were reported in the country through the mandatory surveillance system, 46% of them were infected via injection drug use and 43% through heterosexual contact. The epidemic has not spread to general population and is concentrated in key populations at risk. Half of estimated number of PLHIV in Georgia are unaware of their status [7]. HIV testing activities include community-based testing for key populations at risk (people who inject drugs [PWID] and their sexual partners, men who have sex with men [MSM], female sex workers [FSW]), indicator disease guided testing at selected healthcare facilities, universal testing in antenatal services and mandatory screening of donated blood. Criteria for initiating antiretroviral therapy (ART) have been changing in accordance with evolving international guidelines. Until 2011 ART was recommended at CD4 cell count <200 cells/mm^3^, the threshold was increased to 350 and 500 cells/mm^3^ by 2013, and in 2015 “treat all” policy was implemented in Georgia following the landmark START trial [8].

Previous study conducted in Georgia on late presentation showed that on average 64% of newly diagnosed persons in 2009–2011 presented to care late because of late diagnosis [9]. We aimed to update information on late presentation in Georgia for 2012–2015 to evaluate recent trends, particularly in light of new treatment policies.

## Materials and methods

### Patients and data source

Our analysis included adults (age ≥18 years) newly diagnosed with HIV in Georgia between 1 January 2012–31 December 2015. Persons who did not have CD4 cell count measured within six months of HIV diagnosis were excluded. Person-level data were extracted from the national AIDS Health Information System (AIDS HIS) as of May 1, 2016. AIDS HIS represents secure web-based system connecting all HIV care providers countrywide. It collects information on each reported case of HIV infection, including demographic, epidemiological, clinical and laboratory data.

### Definitions

Late presenter was defined as a person diagnosed with HIV with a CD4 cell count <350 cells/mm^3^ or an AIDS defining illness regardless of the CD4 cell count, in the six months after HIV diagnosis. Late presenter with advanced disease was defined as a person diagnosed with HIV with a CD4 cell count <200 cells/mm^3^or an AIDS defining illness, regardless of CD4 cell count, in the six months after HIV diagnosis [10]. First measurement of CD4 cell count after HIV diagnoses was taken into account.

Patient was defined as entering care, if he/she had documented measurement of CD4 cell count.

For this analysis we divided Georgia into three major regions: 1) capital city of Tbilisi (total population in 2015: 1.1 million people); 2) Western Georgia (total population in 2015: 1.3 million people); 3) Eastern Georgia (total population in 2015: 1.3 million people).

Mode of HIV transmission was assigned to each person at the time of HIV diagnosis by trained professional in accordance with Georgian national surveillance guidelines [11]. This was categorized into four groups: male-to-male sex, injection drug use, heterosexual contact and other, which included cases with undetermined mode of HIV transmission and few cases of blood transfusion related to HIV.

Deaths were attributed to AIDS-related if patient died from AIDS defining illness based on the 1993 CDC classification system [12]. In addition, deaths due to visceral leishmaniasis, Hodgkin’s lymphoma, and non-Hodgkin’s lymphoma of all cell types were classified as AIDS related in accordance with the Coding of Death in HIV (CoDe) protocol [13]. All remaining deaths were classified either as non-AIDS-related causes per CoDe protocol or unknown if information on death was insufficient.

### Statistical analysis

All statistical analyses were performed using SAS statistical software. Proportions of persons presenting late and with advanced disease were calculated for the entire cohort and for each calendar year. Distribution of baseline CD4 cell counts was evaluated for each transmission category. Differences in median CD4 cell count were compared using Mann-Whitney U test. Factors associated with late presentation in univariate analysis were evaluated in multivariate logistic regression.

To evaluate mortality related to late presentation, persons were followed until deaths or May 1, 2016, whichever occurred first. Mortality rates were calculated for the total follow-up period as number of events divided by the number of total person-years of follow-up (PYFU). Kaplan-Meyer analysis was used to estimate probability of survival. Factors associated with mortality were evaluated in multivariate Poisson regression.

Multivariate models were fitted separately for late presenters and late presenters with advanced disease. Factors studied were age at enrolment (per year increase), HIV transmission category, region of Georgia and year of diagnosis.

### Ethical approval

Study was approved by the Institutional Review Board of the Infectious Diseases, AIDS and Clinical Immunology Research Center (OHRP #: IRB00006106). The study used secondary data extracted from the national HIV database as anonymous dataset with no personal identifies, therefore informed consent was waived by IRB.

## Results

A total of 2267 adults were diagnosed with HIV in Georgia in 2012–2015. Among them 1987 (87.6%) had CD4 cell count measured within 6 months of HIV diagnosis and were included in the analysis. Excluded individuals were more likely to be PWID (adjusted odds ratio [aOR]: 2.31, 95% Confidence Interval [CI]: 1.33–4.00), from the capital city Tbilisi (aOR: 1.68, 95% CI: 1.04–2.70) or Western Georgia (aOR: 2.81, 1.80–4.38) and were more likely to die (aOR: 2.30, 95% CI: 1.62–3.26). Overall 59 persons among excluded individuals died, 36 (61.0%) of them died because of AIDS related causes, 13 (22.0%) died from non-AIDS related causes and cause of death could not be established in 10 (16.9%) persons.

Among 1987 persons included in the analysis the median age was 37.1 years and 74.4% were men (Table 1). Half of the study population was infected through heterosexual contact. The number of patients entering care increased from 426 in 2012 to 632 in 2015 (Table 1).

**Table 1:** Factors associated with late presentation and late presentation with advanced disease.

	Total	Presenting late		Presenting late with advanced disease	
		n (%)	aOR (95% CI)	n (%)	aOR (95% CI)
**Total**	1987	1260 (63.4)		870 (43.8)	
**Age at enrolment, median years (IQR)**	37.1 (29.4–44.6)	40.1 (32.6–46.5)	1.06 (1.05–1.07)	41.5 (34.5–47.7)	1.06 (1.05–1.08)
**Sex**					
Men	1478	933 (63.1)	0.94 (0.72–1.22)	655 (44.3)	1.05 (0.81–1.35)
Women	509	327 (64.2)	1	215 (42.2)	1
**HIV transmission category**					
Injection drug use	649	504 (77.7)	2.69 (1.94–3.72)	392 (60.4)	3.17 (2.22–4.55)
Heterosexual contact	1003	623 (62.1)	1.53 (1.12–2.08)	414 (41.3)	1.83 (1.28–2.63)
Other	24	13 (54.2)	0.77 (0.30–1.95)	8 (33.3)	0.75 (0.27–2.07)
Male-to-male sex	311	120 (38.6)	1	56 (18.0)	1
**Region of Georgia**					
Western Georgia	900	627 (69.7)	1.37 (1.10–1.71)	460 (51.1)	1.39 (1.11–1.74)
Eastern Georgia	368	237 (64.4)	1.42 (1.07–1.87)	155 (42.1)	1.26 (0.95–1.66)
Tbilisi	719	396 (55.1)	1	255 (35.5)	1
**Year of HIV diagnosis**					
2012	426	303 (71.1)	1.76 (1.33–2.33)	241 (56.6)	2.34 (1.77–3.08)
2013	424	292 (68.9)	1.75 (1.33–2.32)	206 (48.6)	1.79 (1.36–2.35)
2014	505	314 (62.2)	1.12 (0.87–1.45)	205 (40.6)	1.09 (0.84–1.42)
2015	632	351 (55.5)	1	218 (34.5)	1

A total of 1260 (63.4%) patients were classified as late presenters and 870 (43.8%) as late presenters with advanced disease. The proportion of late presenters declined from 71.1% in 2012 to 55.5% in 2015 (p<0.0001). The proportion of patients presenting late with advanced disease decreased from 56.6% in 2012 to 34.5% in 2015 (p<0.0001).

**Fig 1:**
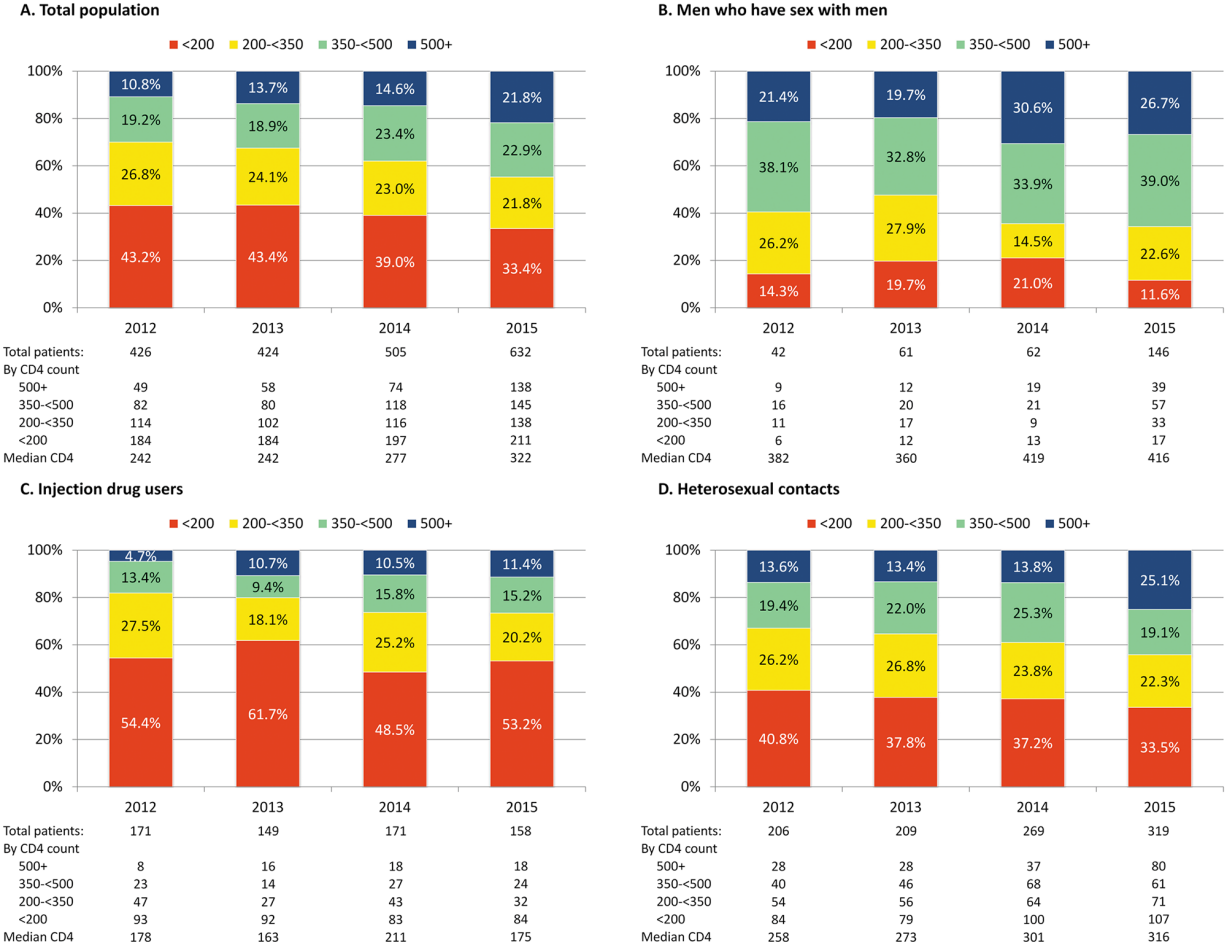
CD4 cell count at the time of HIV diagnosis for total population and HIV transmission categories.

Fig 1 summarizes the distribution of persons by CD4 cell count at the time of diagnosis for total population and disaggregated by transmission category. The number and proportion of persons presenting early with CD4 cell count >500 cells/mm^3^ increased over time from 10.8% in 2012 to 21.8% in 2015 (p<0.0001). The most noticeable increase was seen among MSM and heterosexually infected persons (Fig 1). The median CD4 cell count at the time of diagnosis increased from 242 cells/mm^3^ in 2012 to 322 cells/mm^3^ in 2015 (p<0.0001). The median CD4 cell count at diagnosis did not change significantly over time among MSM and PWID, but increased among heterosexually infected persons from 258 cells/mm^3^ in 2012 to 316 cells/mm^3^ in 2015 (p = 0.009).

Factors associated with late presentation and late presentation with advanced disease included age at enrolment, HIV exposure categories of injection drug use and heterosexual contact as compared to male-to-male sex, and year of HIV diagnosis 2012 and 2013 compared to 2015 (Table 1). Late presenters were more likely to originate from either Western or Eastern Georgia, while only residence in Western Georgia was associated with late presentation with advanced disease.

Patients were followed for median 1.8 (IQR: 0.9–3.0) years and accrued 3929 PYFU. Overall 186 patients (including 171 late presenters and 157 late presenters with advanced disease) died over this period with mortality rate of 4.73 deaths per 100 PYFU (95% CI: 4.10–5.47). The mortality rate was significantly higher both among late presenters (6.74 per 100 PYFU vs. 1.08 per 100 PYFU, p<0.0001) and late presenters with advanced disease (8.93 per 100 PYFU vs. 1.34 per 100 PYFU, p<0.0001).

Of 186 deceased persons 89 (47.8%) died from AIDS related causes, 79 (42.5%) died from non-AIDS related causes and 18 (9.7%) died from unknown causes.

**Fig 2:**
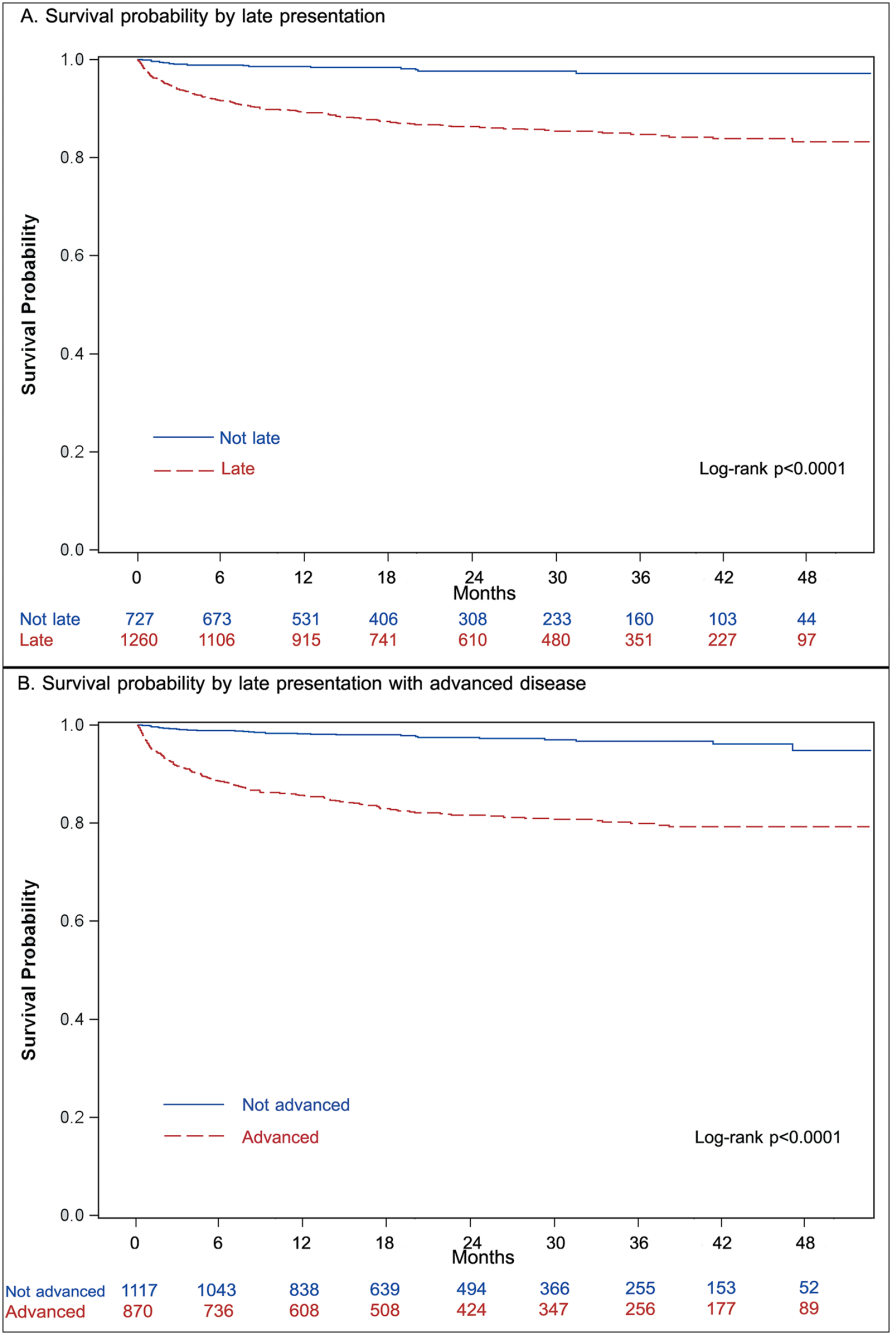
Kaplan-Meier survival curves for late presentation and late presentation with advanced disease.

Kaplan-Meier analysis showed late presenters and those with advanced diseases were more likely to die (Fig 2). The major loss occurred within the year of HIV diagnosis. Survival probability among late presenters dropped to 0.92 (95% CI: 0.90–0.93) at 6 months and to 0.89 (95% CI: 0.87–0.91) at 12 months after HIV diagnosis, while among non-late presenters survival probability remained at 0.98 (95% CI: 0.97–0.99) by 12 months after diagnosis. Survival probability among late presenters with advanced disease was 0.88 (95% CI: 0.86–0.90) by 6 months and 0.85 (95% CI: 0.83–0.87) by 12 months since HIV diagnosis.

Access to ART was high, with 85% of the cohort initiating therapy in median 0.77 months (IQR: 0.43–2.3) after HIV diagnosis. Median time to death among persons not initiating ART was 0.75 months (IQR: 0.34–2.06) after HIV diagnosis.

Factors associated with mortality were assessed in multivariate models separately for late presentation and late presentation with advanced disease (Table 2). Both were significantly associated with the risk of death—late presentation aRR 4.45 (95% CI: 2.59–7.63); late presentation with advanced diseases aRR 4.37 (95% CI: 2.90–6.59). Other factors significantly associated with mortality included not receiving ART, older age at enrolment per year increase, HIV transmission categories of injection drug use and heterosexual contact, earlier year of diagnosis. Association between mortality and residence in Eastern Georgia as compared to capital city showed borderline significance with p values equaling 0.05 in both models. (Table 2).

**Table 2:** Factors associated with mortality.

	Total, n	Died, n (%)	Multivariate model for presenting late	Multivariate model for presenting late with advanced disease
			aRR (95% CI)	aRR (95% CI)
**Total**	1987	186 (9.4)		
**Late presentation**				
Yes	1260	171 (13.6)	4.45 (2.59–7.63)	
No	727	15 (2.1)	1	
**Late presentation with advanced disease**				
Yes	870	157 (18.0)		4.37 (2.90–6.59)
No	1117	29 (2.6)		1
**Age at enrolment, median years (IQR)**	37.1 (29.4–44.6)	44.4 (39.3–51.2)	1.05 (1.04–1.07)	1.05 (1.04–1.06)
**Sex**				
Men	1478	146 (9.9)	1.25 (0.79–1.67)	1.16 (0.81–1.68)
Women	509	40 (7.9)	1	1
**HIV transmission category**				
Injection drug use	649	97 (14.9)	4.30 (1.71–10.79)	4.02 (1.61–10.02)
Heterosexual contact	1003	83 (8.3)	3.15 (1.24–7.99)	2.99 (1.19–7.54)
Other	24	2 (8.3)	2.75 (0.59–12.85)	2.90 (0.63–13.44)
Male-to-male sex	311	4 (1.3)	1	1
**Region of Georgia**				
Western Georgia	900	92 (10.2)	0.86 (0.63–1.17)	0.85 (0.63–1.15)
Eastern Georgia	368	43 (11.7)	1.40 (0.99–1.97)	1.40 (1.00–1.96)
Tbilisi	719	51 (7.1)	1	1
**Year of HIV diagnosis**				
2012	426	52 (12.2)	1.68 (1.13–2.48)	1.73 (1.18–2.54)
2013	424	46 (10.8)	1.72 (1.16–2.57)	1.59 (1.07–2.36)
2014	505	54 (10.7)	1.87 (1.27–2.75)	1.68 (1.15–2.47)
2015	632	34 (5.4)	1	1
**Antiretroviral therapy**				
No	295	62 (21.0)	3.51 (2.70–4.55)	3.18 (2.47–4.09)
Yes	1692	124 (7.3)	1	1

## Discussion

Late presentation of HV infection is a significant public health problem in Georgia, claiming excess number of lives early after HIV diagnosis. Our analysis showed that late presentation peaked in 2012 at 71.1% and decreased to 55.5% in 2015. This was associated with significant increase in CD4 cell count at the time of presentation, even though the median remained below 350 cell/mm^3^.

Decline in late presentation is partially associated with improving HIV testing services in the country over the recent period, including healthcare- and community-based services. New approaches had been implemented since 2012, including adoption of European guidelines “HIV Indicator Conditions: Guidance for Implementing HIV Testing in Adults in Health Care Settings” (available from HIV in Europe website: http://hiveurope.eu/Portals/0/Guidance.pdf.pdf), implementation of incentivized peer-driven intervention for key populations at risk and most recently operation of mobile testing vans for outreach testing. As a result, from 2012 to 2015 HIV testing uptake, defined as receipt of HIV test within 12 months period, improved in all key populations, including among FSW (from 27.5% to 51.8%), MSM (from 25.9% to 44.3%) and PWID (from 5.7% to 25.7%) [14–16]. However, testing uptake remains inadequate and that is why more than half of PLHIV are diagnosed late.

Decrease in late presentation cannot be entirely explained by improved HIV testing strategies, but is also likely to be impacted by increased incidence of HIV infection, particularly among MSM. As HIV incidence increases more people are diagnosed early and hence the proportion of late presenter declines. Of cumulative 5412 cases of HIV reported in the country from 1989 to 2015, 2267 (41.9%) were diagnosed in 2012–2015. Although the number of new HIV cases do not directly measure incidence of infection, increase in new diagnosis can be considered as surrogate marker of increasing incidence. Data from national HIV surveillance and bio-behavioral studies indicate rapid increase in incidence and prevalence of HIV among MSM over the last 5 years, with prevalence now reaching 25% in the capital city of Tbilisi [16, 17]. In our analysis, the number of newly diagnosed MSM increased from 42 in 2012 to 146 in 2015, with almost fourfold increase in the number of persons with CD4 cell count >350 cells/mm^3^. In response to emerging epidemic in MSM, country scaled-up efforts and in addition to standard prevention package, pre-exposure prophylaxis for high risk MSM will become available starting from 2017.

Similar trends of declining late presentation were reported within Collaboration of Observational HIV Epidemiological Research Europe (COHERE) study for the period of 2001–2010, which then stabilized during 2010–2013 at 48–49% [2, 18]. Similar to COHERE study, recent reports from western European countries indicate the prevalence of late presentation of around 50% [19–21]. The data for Eastern European (EE) region is scarce. Data from the European Center for Disease Prevention and Control (ECDC) shows similar rates of late presentation in EE countries ranging from 40.6% to 63.1% [6]. However, two countries with largest HIV epidemics—Russia and Ukraine, did not report data to ECDC, while completeness of reported data for many countries was below 80% [6]. In COHERE the prevalence of late presentation in Eastern Europe was 38.3%, which is significantly lower to that of shown in our study [2]. However, Eastern Europe in COHERE is represented by cohort of patients receiving care at centers of excellence, which may not be representative of the whole region [22].

Important aspect shared by Georgia and entire Eastern European region is the high prevalence of late presentation among PWID. In our analysis 77.7% of people infected through injection drug use presented to care late and 60.4%—with advanced disease. The median CD4 cell count at presentation did not change over time and was 176 cells/mm^3^. As referenced above only 27.5% of PWID received an HIV test in 2015, and the coverage was as low as 5.7% in 2012 [15]. Issue of accessing HIV prevention and care services in Eastern Europe has been well documented previously and this is true for Georgia as well [23]. As in the majority of Eastern European countries drug policy in Georgia is mainly based on strict law enforcement practices, which drives drug users further away from prevention and testing services [24]. Legislative changes will be the key for reversing trends in late diagnosis in PWID.

Interesting finding of our analysis is that people living outside of capital city of Tbilisi are at higher risk for late presentation. This could be related to availability of HIV services, which for many years has been concentrated in the capital. Service coverage has been expanding to regions of Georgia, but as our data suggest further expansion is needed for achieving better results.

It is not surprising that high prevalence of late presentation seen in our analysis resulted in high mortality. Georgia made significant efforts towards improving quality of care through implementing latest European and international guidelines [25]. Package of HIV care services in Georgia includes immediate ART regardless of CD4 cell count, treatment of co-infections such as hepatitis C and tuberculosis, as well as methadone substitution therapy for active drug users. As a result country has highest ART coverage in Eastern European region and HIV related mortality has been declining steadily [1, 26]. However, late diagnosis continues to claim excess number of lives. Both late presentation and late presentation with advanced diseases were major predictors of mortality (aRR4.45 and 4.37 respectively). Mortality was high despite of high ART coverage in the studied cohort with 85% of persons initiating therapy in median 23 days (0.77 months) after HIV diagnosis. This suggest greater impact of late presentation over ART exposure with risk of death being greatest within 6 months of HIV diagnosis, particularly among those presenting late with advanced disease. This is in line with our previous finding indicating dramatic effect of late diagnosis on survival [26, 27] and is also in line with international experience. [18, 28, 29].

Similar to other studies injection drug use was significant predictor of mortality in Georgia [30–32]. The difference was statistically significant with all other transmission categories and this is the result of highest rate of late presentation among drug users. MSM had lowest proportion of persons presenting late and consequently had lowest mortality. Our analysis showed that earlier years of HIV diagnosis compared to 2015 were significantly associated with the risk of death. This temporal association can be related to: 1) increase in the proportion of persons diagnosed earlier and 2) implementation of recommendations towards earlier ART initiation. It also should be taken into account that persons diagnosed in 2015 had shortest follow-up period.

Besides negative impact on clinical outcomes, late presentation has important public health implications in terms of contribution to the growth of the HIV epidemic. Based on seroconverter cohort studies we can assume that nearly 64% of studied population was likely to live with undiagnosed HIV for at least 4 years and potentially were transmitting the virus to others [33]. Recent modeling study showed that more than 90% of new infections can be averted by diagnosing HIV positive persons and engaging them in treatment [34].

Presented work covered all new HIV diagnosis made during 2012–2015 in Georgia, with 87.6% completeness of CD4 cell count data and this provides strengths to our analysis. Major limitation is related to exclusion of 280 persons who had CD4 cell count data missing, which accounted for 12.4% of total new diagnoses. Excluded persons were more likely to be PWID and to die prior to having CD4 cell count measured, thus their exclusion likely resulted in underestimation of late presentation. Despite this limitation, we believe that our analysis provides sufficient power to accurately describe situation on a country level.

In summary, late presentation is the major public health problem in Georgia. Despite the decrease in the proportion of late presenters over time, more than half of newly diagnosed persons enter care late. Late presentation is the main risk factor for mortality and likely also fuels ongoing HIV transmission. Scale-up of HIV testing services, including health sector based and importantly community-based services, is urgently needed. This in turn will require adoption of evidence-based drug policies and eradicating of stigma to establish enabling environment for effective service delivery. Improving earlier HIV diagnosis will be essential step towards ending AIDS epidemic.

## References

[1] UNAIDS. Prevention Gap Report. Geneva: UNAIDS; 2016.

[2] Mocroft A, Lundgren J, Antinori A, Monforte A, Brannstrom J, Bonnet F, et al Late presentation for HIV care across Europe: update from the Collaboration of Observational HIV Epidemiological Research Europe (COHERE) study, 2010 to 2013. Euro Surveill. 2015;20(47). Epub 2015/12/02. doi: 10.2807/1560-7917.ES.2015.20.47.30070 . 26624933 10.2807/1560-7917.ES.2015.20.47.30070

[3] Dickson NP, McAllister S, Sharples K, Paul C. Late presentation of HIV infection among adults in New Zealand: 2005–2010. HIV Med. 2012;13:182–9. doi: 10.1111/j.1468-1293.2011.00959.x 22093231 10.1111/j.1468-1293.2011.00959.x

[4] Zoufaly A, an der Heiden M, Marcus U, Hoffmann C, Stellbrink HJ, Voss L, et al Late presentation for HIV diagnosis and care in Germany. HIV Med. 2012;13(3):172–81. doi: 10.1111/j.1468-1293.2011.00958.x 22093171 10.1111/j.1468-1293.2011.00958.x

[5] Moreno S, Mocroft A, Monforte A. Medical and societal consequences of late presentation. Antivir Ther. 2010;15 Suppl 1:9–15. Epub 2010/05/14. doi: 10.3851/IMP1523 . 20442456 10.3851/IMP1523

[6] European Centre for Disease Prevention and Control, WHO Regional Office for Europe. HIV/AIDS surveillance in Europe 2015 Stockholm: ECDC; 2016.

[7] Chkhartishvili N, Sharavdze L, Chokoshvili O, DeHovitz JA, del Rio C, Tsertsvadze T. The cascade of care in the Eastern European country of Georgia. HIV Med. 2015;16(1):62–6. Epub 2014/06/13. doi: 10.1111/hiv.12172 . 24919923 10.1111/hiv.12172 PMC4264988

[8] Lundgren JD, Babiker AG, Gordin F, Emery S, Grund B, Sharma S, et al Initiation of Antiretroviral Therapy in Early Asymptomatic HIV Infection. N Engl J Med. 2015;373(9):795–807. Epub 2015/07/21. doi: 10.1056/NEJMoa1506816 . 26192873 10.1056/NEJMoa1506816 PMC4569751

[9] Chkhartishvili N, Sharvadze L, Chokoshvili O, Mshvidobadze K, Svanidze M, Gamkrelidze A, et al Late HIV Diagnosis in Georgia: 2009–2011. Tbilisi: Infectious Diseases, AIDS and Clinical Immunology Research Center; WHO Country Office in Georgia; 2012.

[10] Antinori A, Coenen T, Costagiola D, Dedes N, Ellefson M, Gatell J, et al Late presentation of HIV infection: a consensus definition. HIV Med. 2011;12(1):61–4. Epub 2010/06/22. doi: 10.1111/j.1468-1293.2010.00857.x . 20561080 10.1111/j.1468-1293.2010.00857.x

[11] Tsertsvadze T, Imandze P, Baramidze L, Zakhashvili K, Merabishvili T, Burjanadze I, et al Guidellines for Routine Surveillance of HIV/AIDS. Tbilisi: Ministry of Labour, Health and Social Affairs of Georgia; 2009.

[12] Centers for Disease Control and Prevention. 1993 revised classification system for HIV infection and expanded surveillance case definition for AIDS among adolescents and adults. MMWR Recomm Rep. 1992;41(RR-17):1–19. Epub 1992/12/18. . 1361652

[13] Kowalska JD, Friis-Moller N, Kirk O, Bannister W, Mocroft A, Sabin C, et al The Coding Causes of Death in HIV (CoDe) Project: initial results and evaluation of methodology. Epidemiology. 2011;22(4):516–23. Epub 2011/04/28. doi: 10.1097/EDE.0b013e31821b5332 . 21522013 10.1097/EDE.0b013e31821b5332

[14] Tsereteli N, Shengelia N, Sulaberidze L, Chikovani I. HIV risk and prevention behaviours among Female Sex Workers in two cities of Georgia: Bio-behavioral surveillance survey in Tbilisi and Batumi. Tbilisi: Curatio International Foundation; 2014.

[15] Chikovani I, Shengelia N, Sulaberidze L, Sirbiladze T, Tavzarashvili L. HIV risk and prevention behaviors among People Who Inject Drugs in seven cities of Georgia: Bio-Behavioral Surveillance Survey in seven cities of Georgia. Tbilisi: Curatio International Foundation; 2015.

[16] Tsereteli N, Chikovani I, Shengelia N, Sulaberidze L. HIV risk and prevention behavior among Men who have Sex with Men in Tbilisi and Batumi, Georgia: Bio-Behavioral Surveillance Survey in 2015. Tbilisi: Curatio International Foundation; 2015.

[17] Tsertsvadze T, Chkhartishvili N, Dvali N, Karchava M, Chokoshvili O, Tavadze L, et al Estimating HIV incidence in eastern European country of Georgia: 2010–2012. Int J STD AIDS. 2014;25(13):913–20. Epub 2014/03/29. doi: 10.1177/0956462414525939 . 24671716 10.1177/0956462414525939

[18] Mocroft A, Lundgren JD, Sabin ML, Monforte A, Brockmeyer N, Casabona J, et al Risk factors and outcomes for late presentation for HIV-positive persons in Europe: results from the Collaboration of Observational HIV Epidemiological Research Europe Study (COHERE). PLoS Med. 2013;10(9):e1001510 Epub 2013/10/19. doi: 10.1371/journal.pmed.1001510 . 24137103 10.1371/journal.pmed.1001510 PMC3796947

[19] Hachfeld A, Ledergerber B, Darling K, Weber R, Calmy A, Battegay M, et al Reasons for late presentation to HIV care in Switzerland. J Int AIDS Soc. 2015;18:20317 Epub 2015/11/21. doi: 10.7448/IAS.18.1.20317 . 26584954 10.7448/IAS.18.1.20317 PMC4653319

[20] O'Connell S, Enkelmann J, Sadlier C, Bergin C. Late HIV presentation—missed opportunities and factors associated with a changing pattern over time. Int J STD AIDS. 2016 Epub 2016/10/07. doi: 10.1177/0956462416674093 . 27707954 10.1177/0956462416674093

[21] Raffetti E, Postorino MC, Castelli F, Casari S, Castelnuovo F, Maggiolo F, et al The risk of late or advanced presentation of HIV infected patients is still high, associated factors evolve but impact on overall mortality is vanishing over calendar years: results from the Italian MASTER Cohort. BMC Public Health. 2016;16(1):878 Epub 2016/08/26. doi: 10.1186/s12889-016-3477-z . 27557878 10.1186/s12889-016-3477-z PMC4997689

[22] Chene G, Phillips A, Costagliola D, Sterne JA, Furrer H, Del Amo J, et al Cohort Profile: Collaboration of Observational HIV Epidemiological Research Europe (COHERE) in EuroCoord. Int J Epidemiol. 2016 Epub 2016/11/20. doi: 10.1093/ije/dyw211 . 27864413 10.1093/ije/dyw211 PMC6236919

[23] Degenhardt L, Mathers BM, Wirtz AL, Wolfe D, Kamarulzaman A, Carrieri MP, et al What has been achieved in HIV prevention, treatment and care for people who inject drugs, 2010–2012? A review of the six highest burden countries. Int J Drug Policy. 2014;25(1):53–60. Epub 2013/10/12. doi: 10.1016/j.drugpo.2013.08.004 . 24113623 10.1016/j.drugpo.2013.08.004

[24] Otiashvili D, Tabatadze M, Balanchivadze N, Kirtadze I. Policing, massive street drug testing and poly-substance use chaos in Georgia—a policy case study. Subst Abuse Treat Prev Policy. 2016;11(1). doi: 10.1186/s13011-016-0049-2 26772817 10.1186/s13011-016-0049-2 PMC4715284

[25] Kowalska JD, Oprea C, de Witt S, Pozniak A, Gökengin D, Youle M, et al Euroguidelines in Central and Eastern Europe (ECEE) conference and the Warsaw Declaration—a comprehensive meeting report. HIV Medicine. 2016 doi: 10.1111/hiv.12436 27553526 10.1111/hiv.12436

[26] Chkhartishvili N, Sharvadze L, Chokoshvili O, Bolokadze N, Rukhadze N, Kempker RR, et al Mortality and causes of death among HIV-infected individuals in the country of Georgia: 1989–2012. AIDS Res Hum Retroviruses. 2014;30(6):560–6. Epub 2014/01/30. doi: 10.1089/AID.2013.0219 . 24472093 10.1089/aid.2013.0219 PMC4046195

[27] Chkhartishvili N, Sharvadze L, Gabunia P, Abutidze A, Nikolaishvili M, Tsertsvadze T. Late HIV diagnosis in Georgia: public health and economic implications. Translational and Clinical Medicine-Georgian Medical Journal. 2016;1(1):11–4.

[28] May M, Gompels M, Delpech V, Porter K, Post F, Johnson M, et al Impact of late diagnosis and treatment on life expectancy in people with HIV-1: UK Collaborative HIV Cohort (UK CHIC) Study. BMJ. 2011;343:d6016 Epub 2011/10/13. doi: 10.1136/bmj.d6016 . 21990260 10.1136/bmj.d6016 PMC3191202

[29] Sobrino-Vegas P, Moreno S, Rubio R, Viciana P, Bernardino JI, Blanco JR, et al Impact of late presentation of HIV infection on short-, mid- and long-term mortality and causes of death in a multicenter national cohort: 2004–2013. J Infect. 2016;72(5):587–96. Epub 2016/02/28. doi: 10.1016/j.jinf.2016.01.017 . 26920789 10.1016/j.jinf.2016.01.017

[30] Garriga C, Garcia de Olalla P, Miro JM, Ocana I, Knobel H, Barbera MJ, et al Mortality, Causes of Death and Associated Factors Relate to a Large HIV Population-Based Cohort. Plos One. 2015;10(12):e0145701 Epub 2015/12/31. doi: 10.1371/journal.pone.0145701 . 26716982 10.1371/journal.pone.0145701 PMC4696823

[31] Murray M, Hogg RS, Lima VD, May MT, Moore DM, Abgrall S, et al The effect of injecting drug use history on disease progression and death among HIV-positive individuals initiating combination antiretroviral therapy: collaborative cohort analysis. HIV Med. 2012;13(2):89–97. Epub 2011/08/09. doi: 10.1111/j.1468-1293.2011.00940.x . 21819529 10.1111/j.1468-1293.2011.00940.x PMC4539012

[32] Weber R, Huber M, Battegay M, Stahelin C, Castro Batanjer E, Calmy A, et al Influence of noninjecting and injecting drug use on mortality, retention in the cohort, and antiretroviral therapy, in participants in the Swiss HIV Cohort Study. HIV Med. 2015;16(3):137–51. Epub 2014/08/16. doi: 10.1111/hiv.12184 . 25124393 10.1111/hiv.12184

[33] Lodi S, Phillips A, Touloumi G, Geskus R, Meyer L, Thiebaut R, et al Time From Human Immunodeficiency Virus Seroconversion to Reaching CD4+ Cell Count Thresholds <200, <350, and <500 Cells/mm3: Assessment of Need Following Changes in Treatment Guidelines. Clin Infect Dis. 2011;53(8):817–25. doi: 10.1093/cid/cir494 21921225 10.1093/cid/cir494

[34] Skarbinski J, Rosenberg E, Paz-Bailey G, Hall HI, Rose CE, Viall AH, et al Human immunodeficiency virus transmission at each step of the care continuum in the United States. JAMA Intern Med. 2015;175(4):588–96. Epub 2015/02/24. doi: 10.1001/jamainternmed.2014.8180 . 25706928 10.1001/jamainternmed.2014.8180

